# Wee1 promotes cell proliferation and imatinib resistance in chronic myeloid leukemia via regulating DNA damage repair dependent on ATM-γH2AX-MDC1

**DOI:** 10.1186/s12964-022-01021-z

**Published:** 2022-12-27

**Authors:** Fanting Zeng, Yuhang Peng, Yuefeng Qin, Jianming Wang, Guoyun Jiang, Wenli Feng, Ying Yuan

**Affiliations:** 1 Department of Clinical Hematology, Key Laboratory of Laboratory Medical Diagnostics Designated By Ministry of Education, School of Laboratory Medicine, Chongqing Medical University, No. 1, Yixueyuan Road, Yuzhong District, Chongqing, 400016 China; 2 Department of Respiratory and Critical Care Medicine, The First Affiliated Hospital of Chongqing Medical University, No. 1, Youyi Road, Yuzhong District, Chongqing, 400016 China

**Keywords:** Chronic myeloid leukemia, Wee1, DNA damage repair, DNA damage response

## Abstract

**Background:**

The treatment of chronic myeloid leukemia (CML) is facing the dilemma of tyrosine kinase inhibitors (TKIs) resistance and disease recurrence. The dysfunctional DNA damage repair mechanism plays an essential role not only in the initiation and progression of hematological malignancies but also links to the development of TKI resistance. Deciphering the abnormally regulated DNA damage repair and proteins involved brings new insights into the therapy of leukemias. As a G2/M phase checkpoint kinase and a DNA damage repair checkpoint kinase engaged in the DNA damage response (DDR), along with an oncogenic driver present in various cancers, the particular involvement of Wee1 in DNA damage is far from clear. Deciphering its function and targeting it via modulating DNA repair pathways is important for improving our understanding of cancer treatment.

**Methods:**

Wee1 expression was assessed in cell lines using RT-qPCR and western blot, and Wee1 knockdown efficacy was validated using RT-qPCR, western blot, and immunofluorescence. Wee1 function was investigated by CCK-8, colony formation, and flow cytometry assay in vitro. Wee1 role in DNA repair and its interactions with other proteins were then studied using western blot, immunofluorescence, and double plasmid-repair studies. Finally, the CCK-8 and flow cytometry assay was utilized to investigate Wee1 and imatinib’s synergistic effect, and a CML mouse model was constructed to study Wee1’s role in carcinogenesis in vivo.

**Results:**

Wee1 was reported to respond quickly to DDR in an ATM-γH2AX-MDC1-dependent way upon DNA double-strand breaks (DSBs) occurrence, and it regulated homologous recombination by stimulating the recruitment of critical proteins RAD51/BRCA1 upon DSB sites. Wee1 was also revealed to be abnormally upregulated in CML cells. Further suppression of Wee1 not only causes cell cycle arrest and inhibits the proliferation of cancer cells but also enhances CML cell sensitivity to Imatinib in vitro and in vivo, possibly through an excessive accumulation of overall DSBs.

**Conclusion:**

Wee1 is extensively involved in the DRR signaling and DSB repair pathway. Inhibiting abnormally elevated Wee1 benefits CML therapy in both IM-resistant and IM-sensitive cells. Our data demonstrated that Wee1 participated in promoting cell proliferation and imatinib resistance in chronic myeloid leukemia via regulating DNA damage repair dependent on ATM-γH2AX-MDC1. In the fight against CML, Wee1’s dysregulation in the DNA damage repair mechanism of CML pathogenesis makes it a viable therapeutic target in clinical applications.

**Supplementary Information:**

The online version contains supplementary material available at 10.1186/s12964-022-01021-z.

## Background

Chronic myeloid leukemia (CML) is a type of stem cell-derived hematopoietic cancer marked through a t (9;22) (q34;q11) chromosome translocation that gives rise to the oncogenic fusion gene bcr-abl. BCR-ABL fusion protein has considerably enhanced tyrosine kinase activity, which is the major cause of CML [1]. Imatinib (IM), a well-known tyrosine kinase inhibitor (TKI), has served as the primary clinical therapy for decades. However, IM drug resistance and intolerance remain an issue in a certain portion of individuals, rendering leukemia stem cells ineffective and contributing to recurrence after treatment discontinuation [2]. In terms of the disadvantages, it is necessary to develop alternative CML treatment methods.

Dysregulated DNA damage repair mechanism is critical in the development of both solid tumors and hematological malignancies [3]. Accumulating shreds of evidence suggest that an abnormally high capacity of DNA repair is linked to CML malignancy, allowing CML cells to survive by escaping cytotoxic drug-induced genome damage. Targeting the DNA damage response (DDR) pathway or combining treatment with chemotherapy provide a viable approach for some leukemias [4, 5]. In mammalian cells, when DNA double-strand breaks (DSBs) occur, the DNA repair mechanism is predominantly started by the DDR, in which the ataxia-telangiectasia kinase (ATM) plays a critical function [6]. ATM is activated instantly by the MRE11-RAD50-NBS1 complex [7] and attracts the mediator of DNA damage checkpoint protein 1 (MDC1) by phosphorylating Ser139 (γH2AX) of chromatin bordering the DSBs of the H2AX tail, generating a complicated feedback loop that leads to γH2AX amplification and stability, and thus further developing a framework for the recruitment and accumulation of a bundle of key DNA repair components [8, 9]. Subsequently, DSB repair can be therefore carried out through homologous recombination (HR) and non-homologous end joining (NHEJ) to restore genomic integrity and promote cell survival [10, 11]. Therefore, the dysregulated DDR pathways associated with CML, as well as the proteins involved, need further investigation for exploring potential therapeutic strategies.

As a serine/threonine protein kinase, Wee1 is a DNA damage repair checkpoint kinase as well as a G2/M phase checkpoint kinase [12, 13]. When the ataxia telangiectasia and RAD3-related (ATR) and ATM signaling pathways of DDR are activated following DSBs, ATM stimulates the downstream checkpoint kinase CHK2, whereas ATR stimulates checkpoint kinase CHK1, and the two checkpoint kinases cooperate to activate Wee1. Once activated, Wee1 primarily inhibits CDK1 activity by phosphorylating the CDK1 Tyr15 site, preventing cells from entering the M phase and causing the mitotic delay, and inducing DNA replication [14–18]. Upregulation of Wee1 is not only associated with cancerogenesis including hepatocellular carcinoma [19], breast cancer, lung cancer [20], and leukemia [21, 22] but also related to tumor progression, poor disease-free survival, and worse prognosis [23–27]. Notably, Wee1 overexpression is a chemotherapy adaptive response that allows cancer cells to enhance DNA damage repair and thus survival, implying Wee1 is a critical function in the DNA damage repair process [28]. Inhibiting Wee1 kinase not only boosts chemotherapeutic drug sensitivity but also forces tumor cells into mitosis, even if the DNA is damaged. Consequently, this eventually leads to cell death and mitotic catastrophe [5]. Wee1 deficiency results in increased H2AX phosphorylation and widespread DDR activation [29]. Although Wee1 is primarily considered to conduct an oncogenic function in CML based on works of literature and previous researchers have correlated Wee1 to DNA damage mechanism, the particular regulation of Wee1 in DNA damage repair and DDR is far from clear in CML. Hence, investigating its function and targeting it via regulating DNA repair pathways is important for CML treatment in addition to the commonly used tyrosine kinase inhibitors.

Wee1 responded to DDR in an ATM-γH2AX-MDC1-dependent way, and it also enhanced the recruitment of essential HR repair proteins RAD51/BRCA1 upon DSBs, according to our findings. Wee1 was highly expressed in CML cells. Additionally, inhibiting Wee1 not only triggers cell cycle arrest and suppresses the development of cancer cells but also enhances CML cell sensitivity to IM in vitro and in vivo. Accordingly, targeting DDR damage proteins like Wee1 and modulating DRR signaling and the DSB repair pathway provided a fresh perspective into carcinogenesis and cancer treatment, notably in hematological malignancies where the dysfunctional DNA damage repair mechanism is highly involved.

## Materials and methods

### Cell lines

293T, SUP-B15 cells in DMEM (Gibco, USA). K562/G01, KCL22, K562, THP1, and TK6 cells were cultured in RPMI-1640 (Gibco, USA). Both media were supplemented with 10% fetal bovine serum. All cells were maintained at 37 °C in an incubator with humidified 5% CO_2_ gas. The Chinese Medical Science Academy’s Cell Culture Center in Shanghai provided cell lines for this study.

### Chemicals and antibodies

The relevant antibodies are utilized for western blots or immunofluorescence: anti-Wee1, anti-γH2AX (CST, USA), anti-MDC1, anti-ATM (Beyotime, China), anti-BRCA1 (Proteintech, China), anti-RAD51 (Wanleibio, China). Among the major inhibitors used were ATM inhibitor, and Imatinib (TargetMol, USA), Calicheamicin-γ1 (MCE, USA) which were all dissolved in DMSO to a concentration of 10 Mm and kept at − 20 °C.

### Infection with lentiviral

Genechem (Shanghai, China) produced and packaged the NC (shNC) and Wee1 shRNAs (shWee1). At a multiplicity of infection (MOI) of 30, the lentivirus was transfected into the K562, K562/G01, and KCL22 cells, which were plated at a density of 2 × 10^5^ cells per well in 6-well plates, and given 2 μg/ml puromycin therapy (Sigma, USA). After a 72h infection period, the changes in the cells in vitro were assessed. To assess the function of IM on CML cell lines infected with shWee1, cells were exposed to various dosages of IM for an additional 2 days before being collected for further study.

### Quantitative real-time PCR (RT-qPCR)

For RT-qPCR, RNA extraction and reverse transcription into cDNA. Takara Bio supplied the chemicals and industry-standard techniques. Additional file 1: Table S1 displays the primer sequences.

### Western blot

Cells were lysed using a RIPA lysis mixture that contained proteinase and phosphatase inhibitors. The extracts were then wet-transferred onto PVDF membranes (Millipore, MA) and separated using SDS-PAGE (8–10% sodium dodecyl sulfate–polyacrylamide gel electrophoresis). Western blotting substrate known as super ECL plus was employed to perform detection (Baoguang, China).

### Alkaline comet assay

Cells were harvested and blended with 0.5% low melting point agar before being put on 1% normal melting point agar-coated slides. After a ten-minute cooling period at 4 °C, the product is ready to use. The slides were lysed for 1 h in the dark at 4 °C before being submerged in an electrophoresis buffer for 20 min. The slides were stained with EB solution before being viewed with a fluorescence microscope. The DNA tail percent was estimated by using the CASP software tool.

### HR and NHEJ reporter assay

The pimEJ5GFP or pDRGFP plasmids (Addgene, USA) were employed to transfect cells with the lipo8000 Reagents (Beyotime, China), and puromycin screening was used to ensure that the DR-GFP construct was stably expressed. Then, using lipo8000 reagents, the pCBASceI expression vector was transiently transfected (Addgene, USA). The FACS Vantage SE system was applied to measure the GFP signal 48 h after transfected (BD Biosciences).

### Immunofluorescence assay

Cells were collected, fixated in 4% paraformaldehyde for 30 min at 37 °C, penetrated with 0.1% Triton X-100 for 15 min, and then blocked with 1% BSA for an hour at 37 °C. At 4 °C, cells were treated with primary antibodies overnight (1:100 in 5% goat serum). The cells were then incubated for an hour in the darkness at 37 °C with a fluorescent secondary antibody. The nuclei were stained with DAPI (4,6-diamidino-2-phenylindole).

### Cell viability assay

In 96-well plates, 3000 cells per well were planted and incubated at 37 °C in an incubator under humidified 5% CO2. At the specified time, 10 μl cell counting kit-8 (CCK-8) (Topscience, China) was got to add per each well and at 37 °C for 2 h. At 450 nm, absorption values were measured.

### Colony-forming assay

At a density of 100 cells per well, the treated cells were counted and implanted on 96-well plates. Using an inverted microscope, the colonies were counted 7 days later.

### Apoptosis and cell cycle analysis

After washing the cells in PBS and adjusting the cell concentration to 2 × 10^6^/mL, the Annexin PE/7-AAD Apoptosis Detection Kit (Vazyme, China) was employed under the manufacturer’s instructions. 1 × 10^6^ cells were rinsed using PBS and fixed in 75% ethanol for the duration of the cell cycle assay. Cell cycle and apoptosis were measured through Coulter FC500 flow cytometry (Beckman, USA).

### Murine tumor model

K562/G01 cells infected with lentivirus were harvested, and the concentration was maintained at 2.5 × 10^7^/ml. Five-week-old female NOD/SCID mice received 250cGy radiation before intravenous injection. Twenty-four mice were separated into four groups at random, with each group receiving 200 μl of cells. Mice were inoculated for 7 days, then given an intraperitoneal injection of IM (50 mg/kg) for 3 weeks. The control was PBS. These mice’s body weights and the quantity the white blood cells in their blood were monitored once every week. Chongqing Medical University’s Ethics Committee authorized all of the experiment’s procedures.

### Statistical analysis

The mean ± SD was used to express all statistical findings. To analyze the statistical differences between student’s t-tests (two groups) and one-way ANOVA (three or more groups), GraphPad Prism 8.0 was used. Statistics were deemed significant at *p* < 0.05.

## Results

### Knocking down abnormally overexpressed Wee1 influences CML cell proliferation and causes cell cycle arrest

**Fig. 1:**
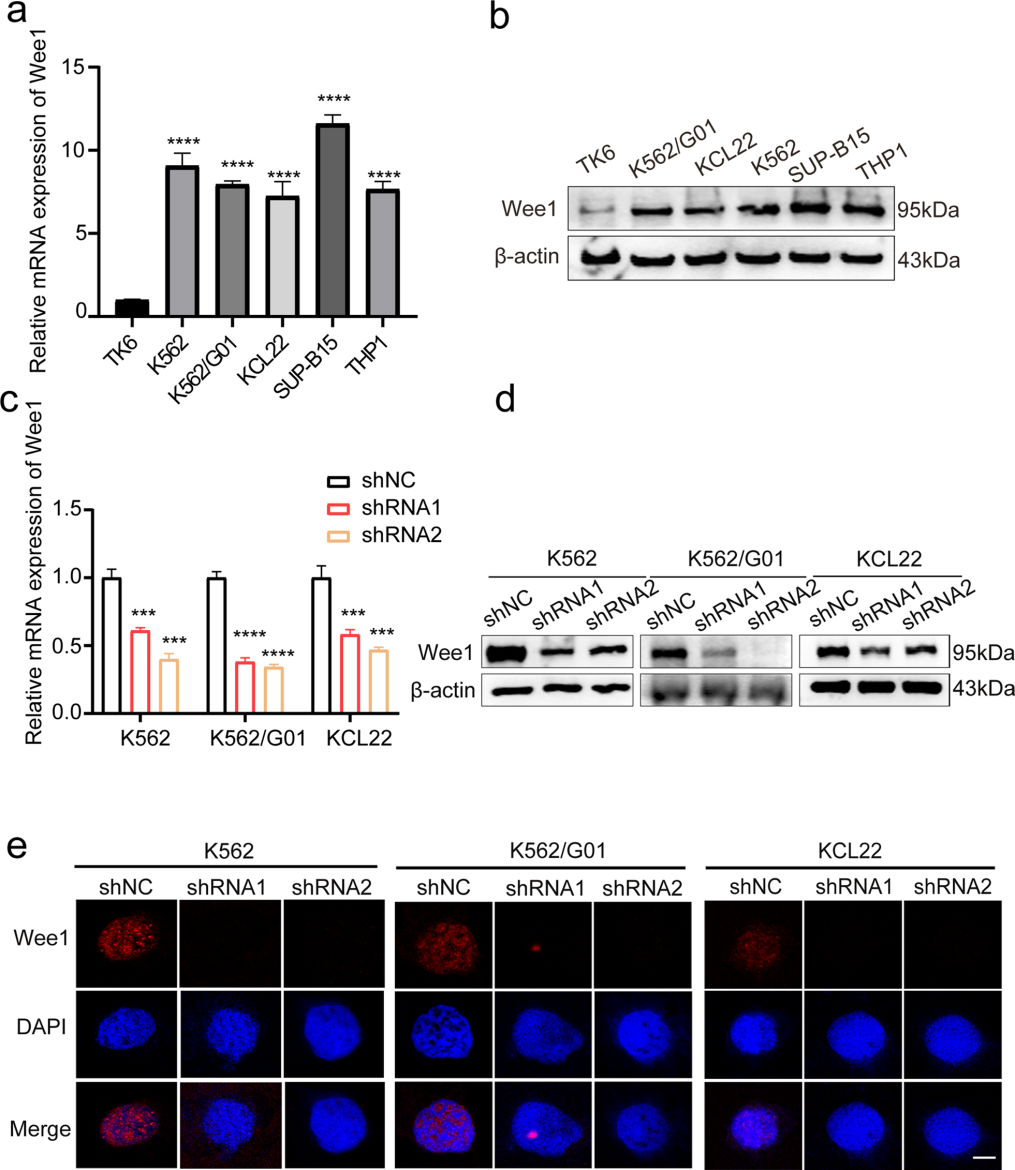
Expression of Wee1 in various leukemic cell lines. **a** Wee1 mRNA expression was detected in leukemia cells SUP-B15, THP1, K562, K562/G01, KCL22, and TK6. **b** Wee1 protein levels were measured in leukemia cells SUP-B15, THP1, K562, K562/G01, KCL22, and TK6. The effect of shRNA knockdown was confirmed using RT-qPCR (**c**), western blot (**d**) and immunofluorescence analysis (**e**). Scale bar, 10 μm. ***p < 0.001, ****p < 0.0001

**Fig. 2:**
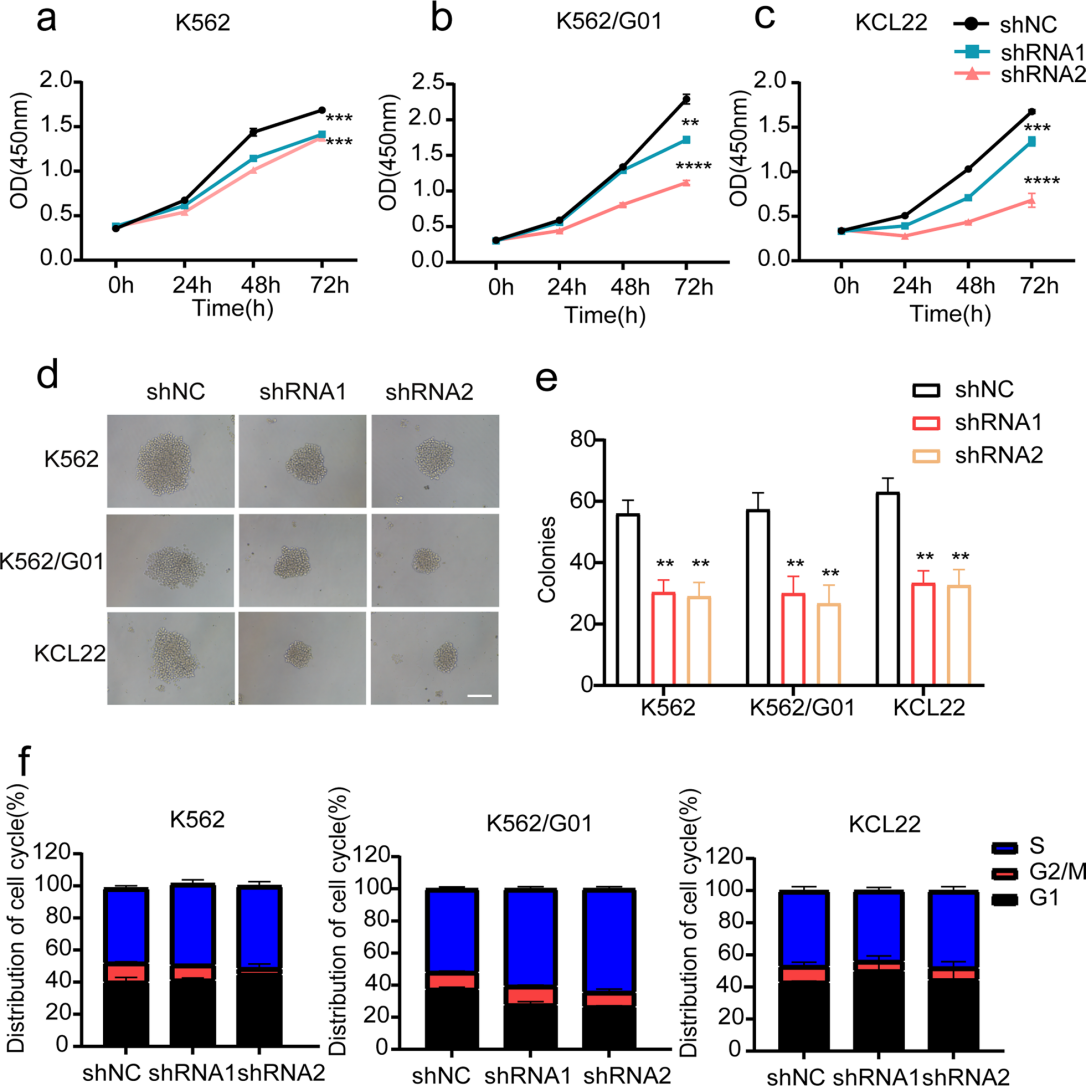
Wee1 silencing influences CML cell proliferation and induces cell cycle arrest. **a**–**c** To calculate the OD values of Wee1 knockout CML cells, CCK-8 assays were employed. In lentivirus-infected K562, K562/G01, and KCL22 cells, the size (**d**) and quantity (**e**) of colonies were counted. **f** Wee1 silencing cells’ cell cycle was investigated through flow cytometry. **p < 0.01, ***p < 0.001, ****p < 0.0001

We first investigated Wee1 expression on CML cells and discovered that Wee1 was upregulated in leukemia cell lines compared to TK6 in terms of both mRNA and protein expression (Fig. 1a, b). The levels of Wee1 in K562, K562/G01, and KCL22 cells were then downregulated using shRNAs and the silencing efficacy was assessed using RT-qPCR, western blot, and immunofluorescence shown in Fig. 1c–e. The RT-qPCR results showed that shWee1 decreased Wee1 expression dramatically, which was later confirmed by the western blot and immunofluorescence. To explore the effect of Wee1 in CML pathogenesis, we found that Wee1 knockdown was associated with reduced CML cell growth (Fig. 2a–c), as well as smaller (Fig. 2d) and fewer (Fig. 2e) cell colonies formation, and induced cell cycle arrest shown by PI staining (Fig. 2f). These data implied that targeting Wee1 affects both CML cell proliferation and cell cycle regulation, thus revealing anticancer manifestations and potential clinical applications, which is presumably due to its involvement in the DNA damage response mechanism according to our hypothesis.

### Wee1 suppression results in the enhancement of the overall DNA damage level

**Fig. 3:**
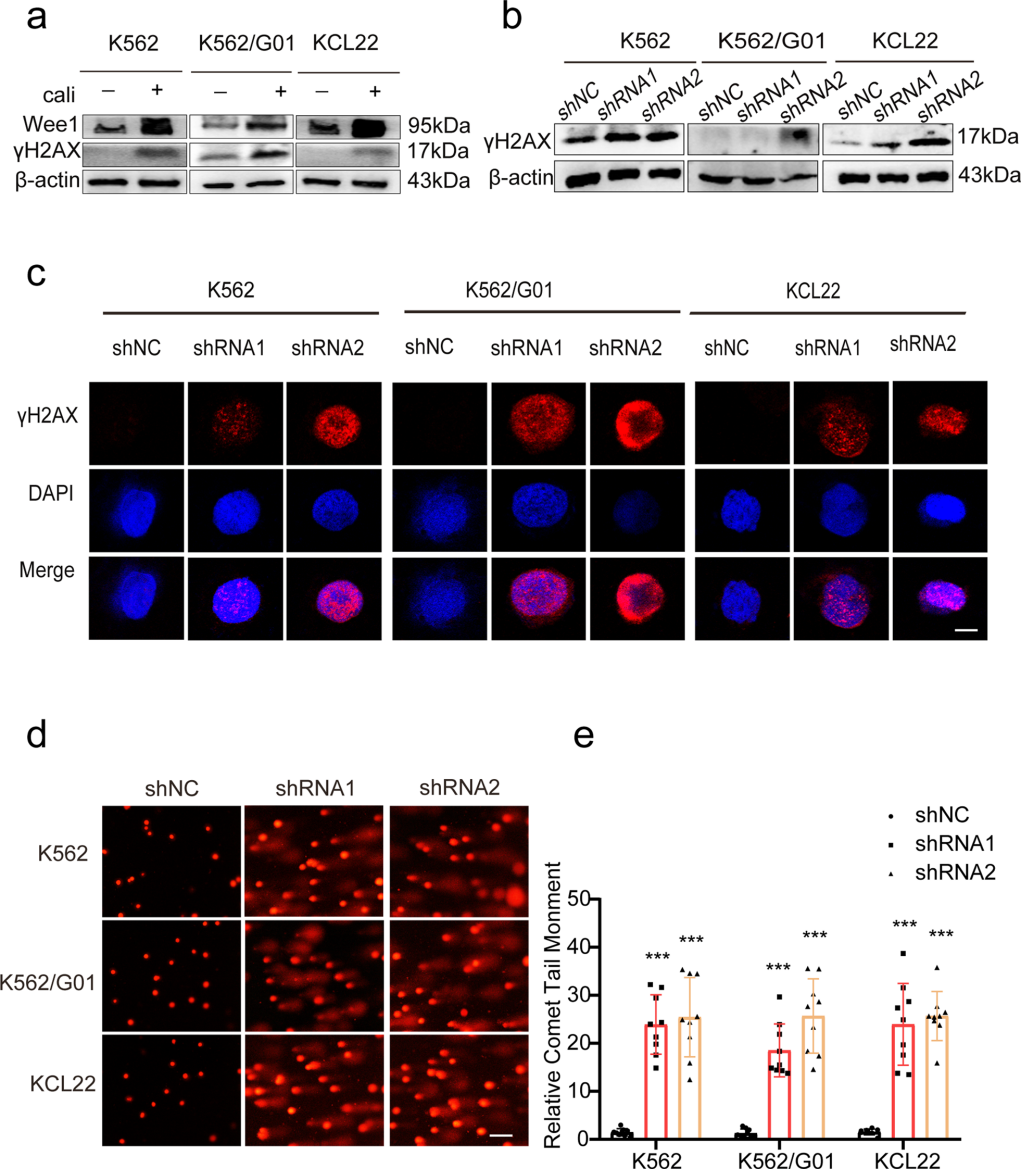
DNA damage is activated as a result of Wee1 deficiency. **a** 44 nM Cali was applied to CML cells for 1 h, we used a western blot assay to determine how Wee1 and γH2AX levels had changed. **b** The level of γH2AX in Wee1 knockdown cells was determined using a western blot. **c** The accumulation of γH2AX in Wee1 knockdown cells is confirmed by immunofluorescence. Scale bar, 10 μm. **d**, **e** A comet assay was used to detect DNA damage. The comet tail moment of 10 cells in each group was calculated using the CASP program. ***p < 0.001

To further look into Wee1’s role in DDR contributed to the anticancer effect of CML, Wee1 expression in CML cells was first assessed by western blot following the DSBs induced by the DNA damage agent calicheamicin (Cali). We observed that the expression of Wee1 and γH2AX rapidly increased (Fig. 3a), suggesting Wee1’s response to DNA damage, consistent with the previous studies [12, 13]. To verify whether the anticancer effects were related to enhanced DNA damage activity, we examined the interplay between γH2AX and Wee1. We found that Wee1 knockdown significantly augmented γH2AX expression (Fig. 3b). Immunofluorescence analysis also confirmed the result (Fig. 3c). In addition, the comet tail substantially increased following Wee1 knockdown in comet assay (Fig. 3d, e), indicating that suppressing Wee1 in CML cells results in the enhanced accumulating of more unrepaired DNA damage, thus explaining the anticancer manifestations.

### *Wee1 responds to DNA damage *via* an ATM-γH2AX-MDC1-dependent pathway*

**Fig. 4:**
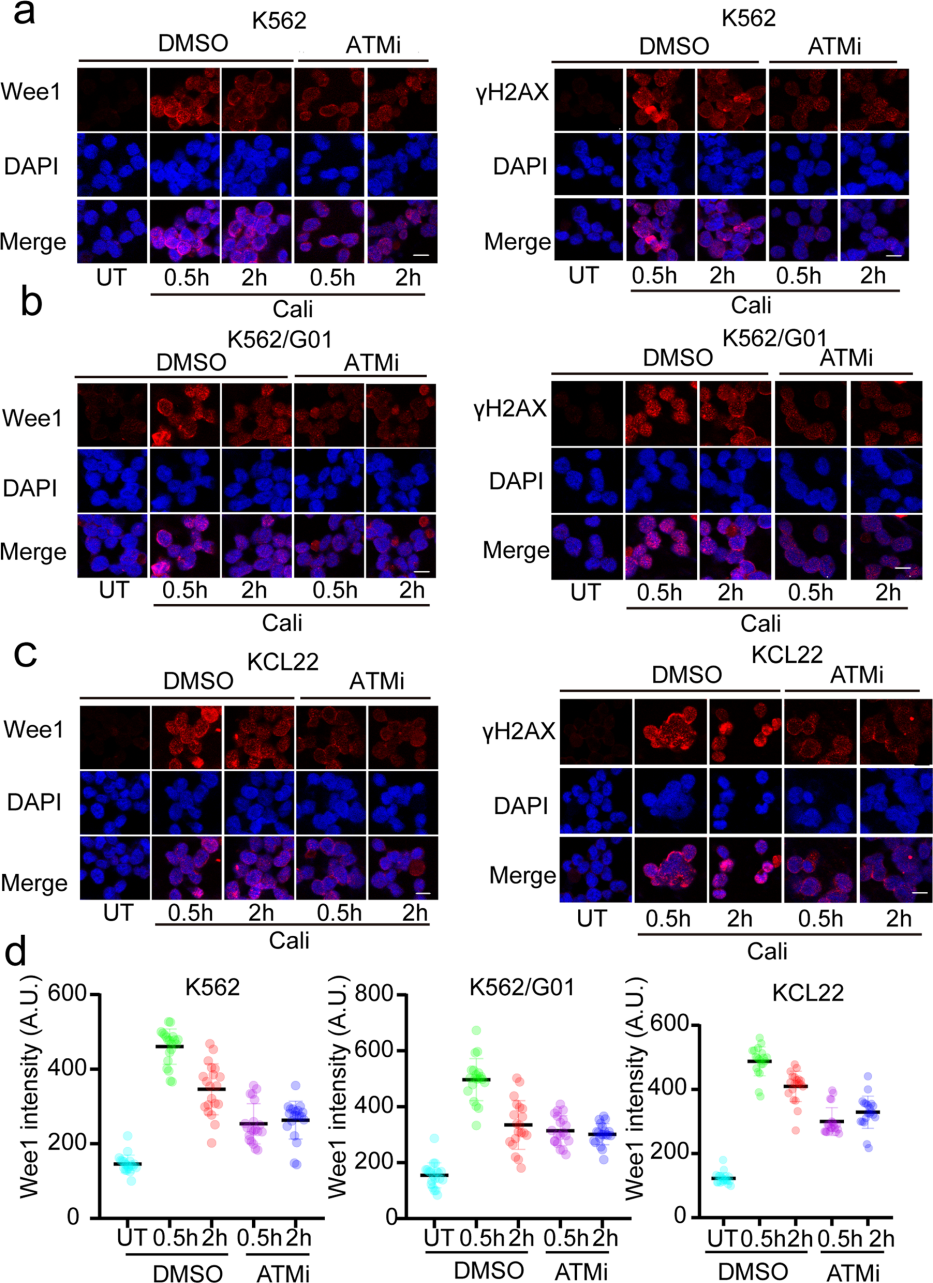
Upon activation of DNA damage, Wee1 recruitment is stimulated by ATM signals. **a–c** Immunofluorescence evaluation of DNA damage, nuclear Wee1 localization, and γH2AX in CML after inhibitor therapy (10 μM, 12 h), Cali treatment (44 nM Cali), or no treatment. **d** The nuclear intensity of Wee1 is represented by a scatter dot plot. Scale bar, 50 μm

**Fig. 5:**
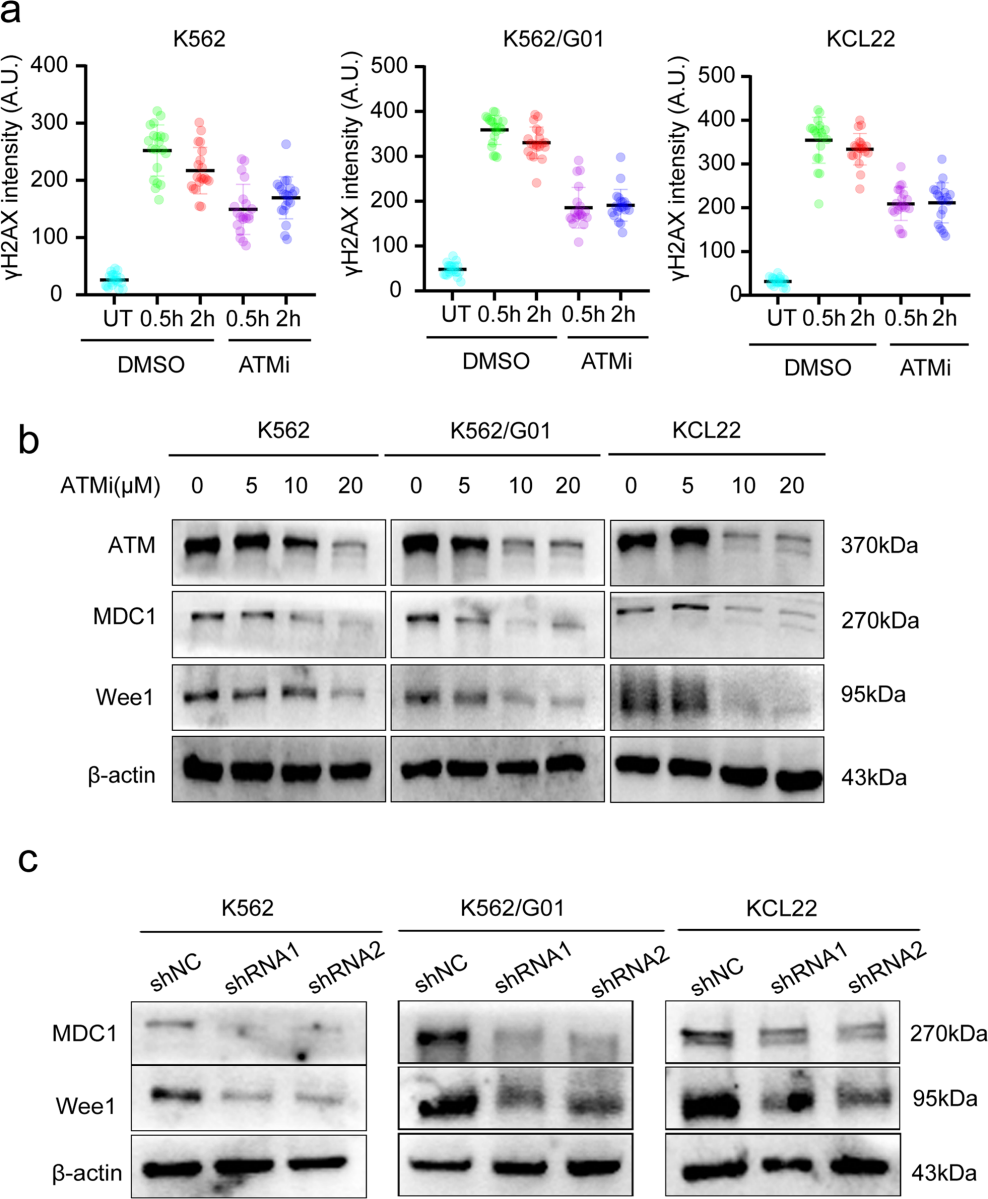
Wee1 reacts to DNA damage via ATM-γH2AX-MDC1. **a** The nuclear intensity of γH2AX is represented by a scatter dot plot. **b** Different doses of ATM inhibitors were applied to CML cells for 24 h and then subjected to western blot analysis. **c** MDC1 and Wee1 expressions were detected in MDC1 silencing cells

The confirmation of Wee1’s association with the DDR signaling pathway now allows us to further explore how Wee1 extensively participates in the DDR and supports DNA damage repair process. To investigate Wee1’s function in DDR in better detail, Wee1’s location and dynamic change were first determined via immunofluorescence after DSBs generated by a DNA damage inducer. We observed an obvious increase in Wee1 accumulation in the nucleus which peaked at 0.5 h and accompanied the generation of phosphorylation H2AX (γH2AX) (Figs. 4a–d, 5a). Moreover, in the early DDR, Wee1 is shown to be recruited since DNA damage agent-induced surge in this protein was dynamic and reduced after 2 h whereas the γH2AX level remained still high (Figs. 4a–d, 5a). Then, to clarify the underlying mechanism linked to this rapid accumulation of Wee1, CML cells were pretreated to ATM inhibitors of the upstream DDR kinase before exposure to a DNA-damaging agent. Interestingly, Wee1 and γH2AX focal accumulation were found halted when ATM, the primary transducer in DDR, was inhibited (Figs. 4a–d, 5a). Subsequently, we used an ATM inhibitor and shRNA repressing MDC1 to better understand the signaling pathways downstream of ATM that regulate Wee1 recruitment. The result suggested that Wee1 and MDC1 expression levels have somewhat decreased as a result of blocking ATM (Fig. 5b) and that knocking down MDC1 will also result in a drop in Wee1 expression (Fig. 5c). We, therefore, demonstrated that Wee1 responds to DNA damage signaling pathway via an ATM-γH2AX-MDC1 dependent manner.

### Wee1 regulates homologous recombination through recruiting RAD51 and BRCA1

**Fig. 6:**
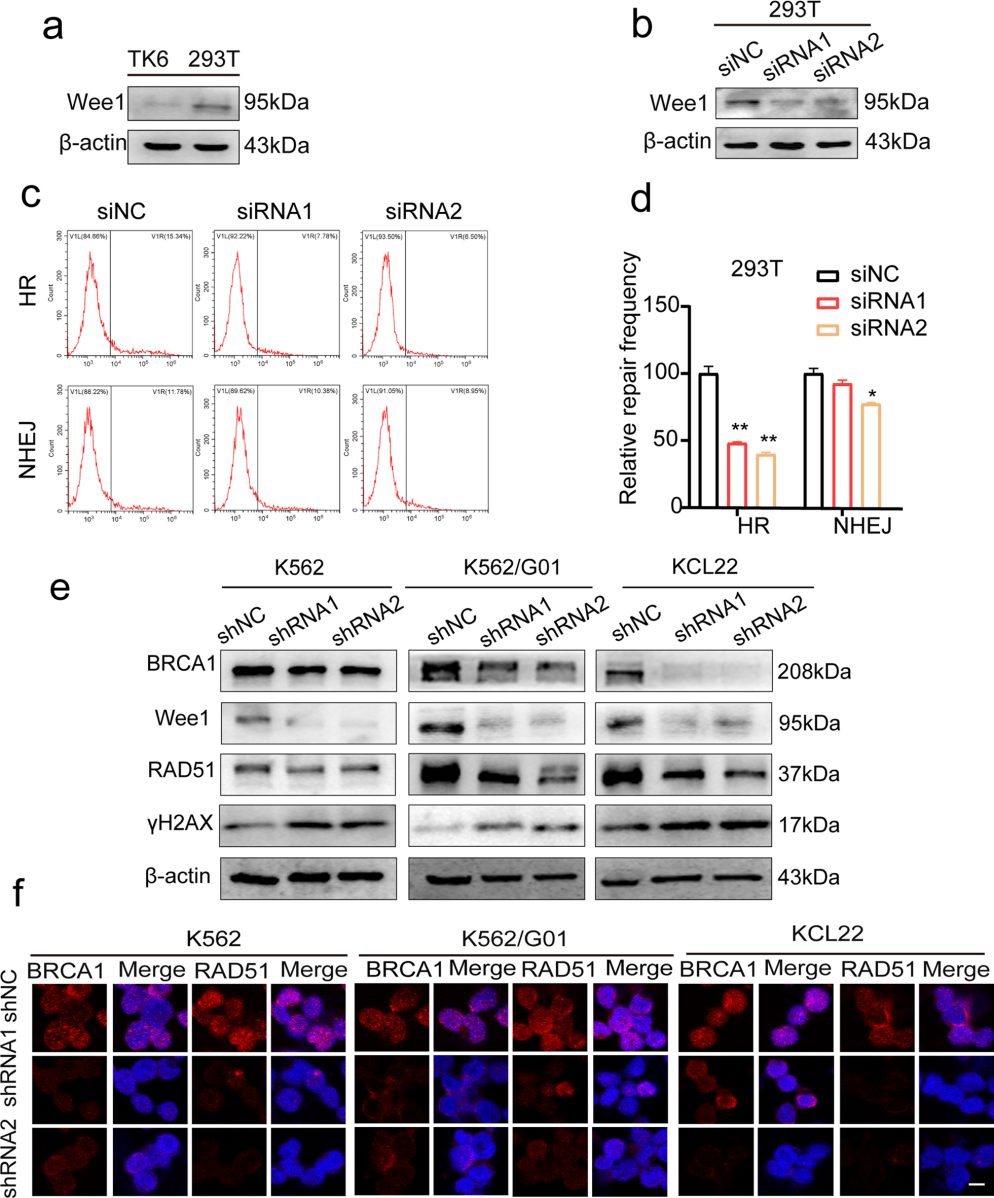
Wee1 binds to DDR proteins and controls cell HR pathways. **a** Wee1 expression in 293T cells was explored using western blot. **b** Western blot analysis of siRNA silencing efficiency. **c**, **d** In the siNC group and siWee1 group, the ratio of GFP^+^ indicated HR or NHEJ repair activity. **e** Western blot and immunofluorescence (**f**) were used to confirm DDR protein (BRCA1, RAD51) expression. Scale bar, 50 μm. *p < 0.05, **p < 0.01

After confirming Wee1’s critical role in DDR and its interaction with certain essential DDR signaling proteins, we tried to explore its modulation related to the major DSB repair pathways downstream and their key proteins interacted. To learn more about Wee1’s role in the DNA repair pathway, the reporter plasmids EJ5-GFP and DR-GFP were used to build up the double vector system in 293T cells to evaluate the DNA repair pathway efficiency respectively following Wee1 knockdown, as previously described [30]. Wee1 is discovered to be substantially expressed in 293T cells (Fig. 6a), and siRNA was used to knock it out. (Fig. 6b) and the double plasmid-repair experiment was performed *in cellulo* (Fig. 6c). HR repair activity was dramatically reduced upon Wee1 was knocked down by two distinct siRNAs, while NHEJ ligation activity was somewhat reduced (Fig. 6d). Given that RAD51 and BRCA1 are essentially required for a successful HR repair [31], we noticed that Wee1 knockdown also compromised the protein expression and inhibited the hiring of RAD51/BRCA1 towards the DNA damage site (Fig. 6e, f). According to the findings, Wee1 knockdown could impair overall intracellular DSB repair activity, particularly HR, and that Wee1 is required for RAD51 and BRCA1 recruitment for functional HR repair, indicating its importance in not only responding to DDR but also communicating downstream DSB repair via key repair proteins.

### *Targeting Wee1 enables CML cells to become more sensitive to IM *in vitro* and *in vivo

**Fig. 7:**
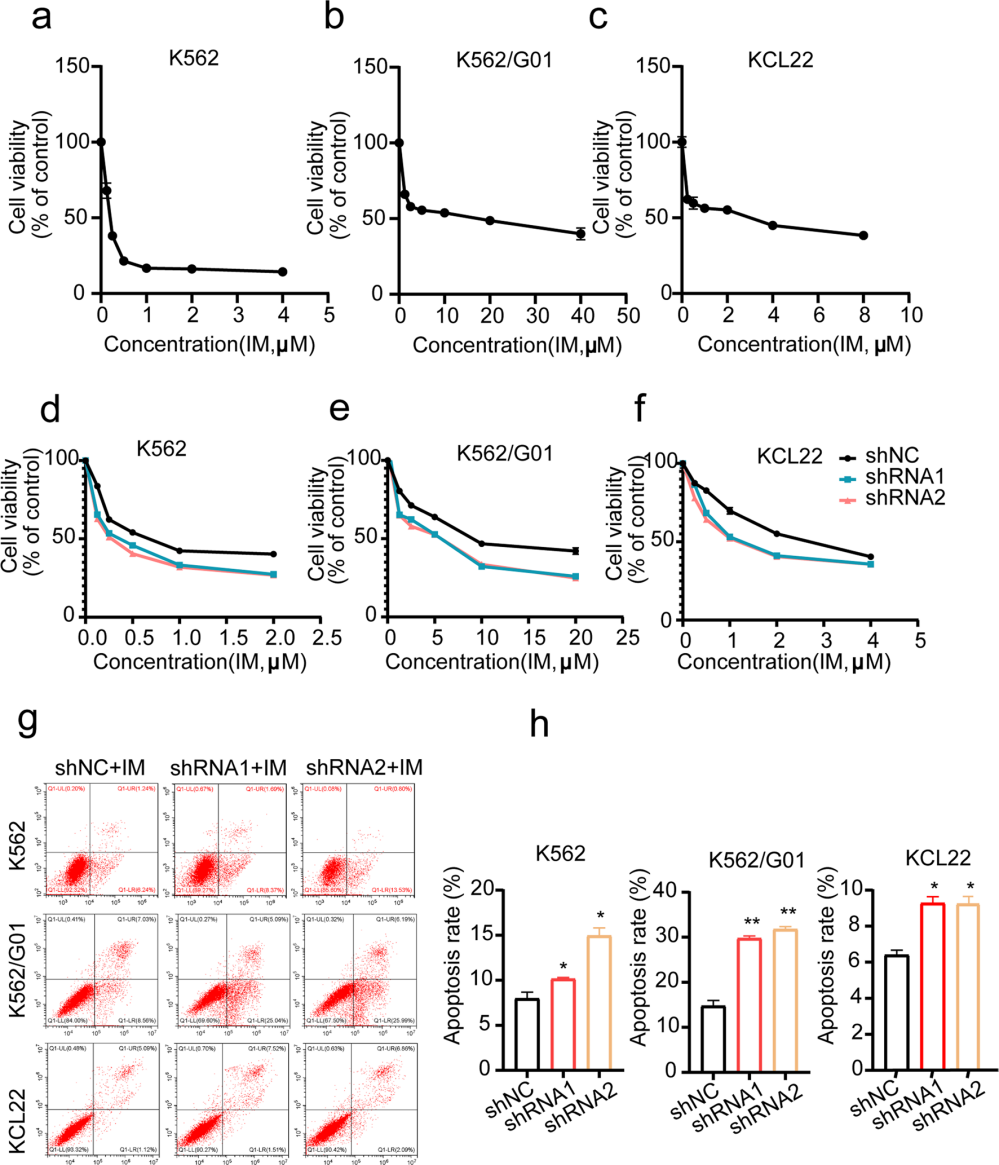
Wee1 silencing improves the influence of IM on Cell lines. **a–c** The CCK-8 assays were utilized to calculate the IC50 value of CML cells treated with a gradient IM concentration for 48 h. **d–f** Drug sensitivity assay of Wee1 knockdown in CML cells. **g**, **h** ShWee1 and shNC were given IM for 48 h (K562 and KCL22 = 0.5 μM, K562/G01 = 5 μM). Apoptosis was detected by FCM. *p < 0.05, **p < 0.01

It has been widely accepted that dysregulated DNA damage repair occurs quite frequently in hematological malignancies, attributed to their pathogenesis, disease progression, and drug resistance. Pieces of evidence have also shown that dysfunctional DNA repair is a possible factor for bcr-abl fusion genetic mutations, and the IM resistance mechanism has been linked to such mutations in CML to some extent [32]. Our results have shown that suppression of Wee1 impaired DSB repair ability in CML cells, we wondered if it could be related to IM sensitizing. Firstly, the IC50 of CML cell lines was calculated (Fig. 7a–c). The CCK8 assay demonstrated that Wee1 knockdown significantly affected the IC50 in CML cell lines when treated with different concentrations of IM (Fig. 7d–f), implying that Wee1 knockdown increased CML cells’ sensitivity to IM. Flow cytometry analysis revealed that Wee1 knockdown rendered CML cell lines to be more vulnerable to IM-induced apoptosis (Fig. 7g, h). These findings suggested that targeting Wee1 could be used to improve IM sensitivity in CML cells.

**Fig. 8:**
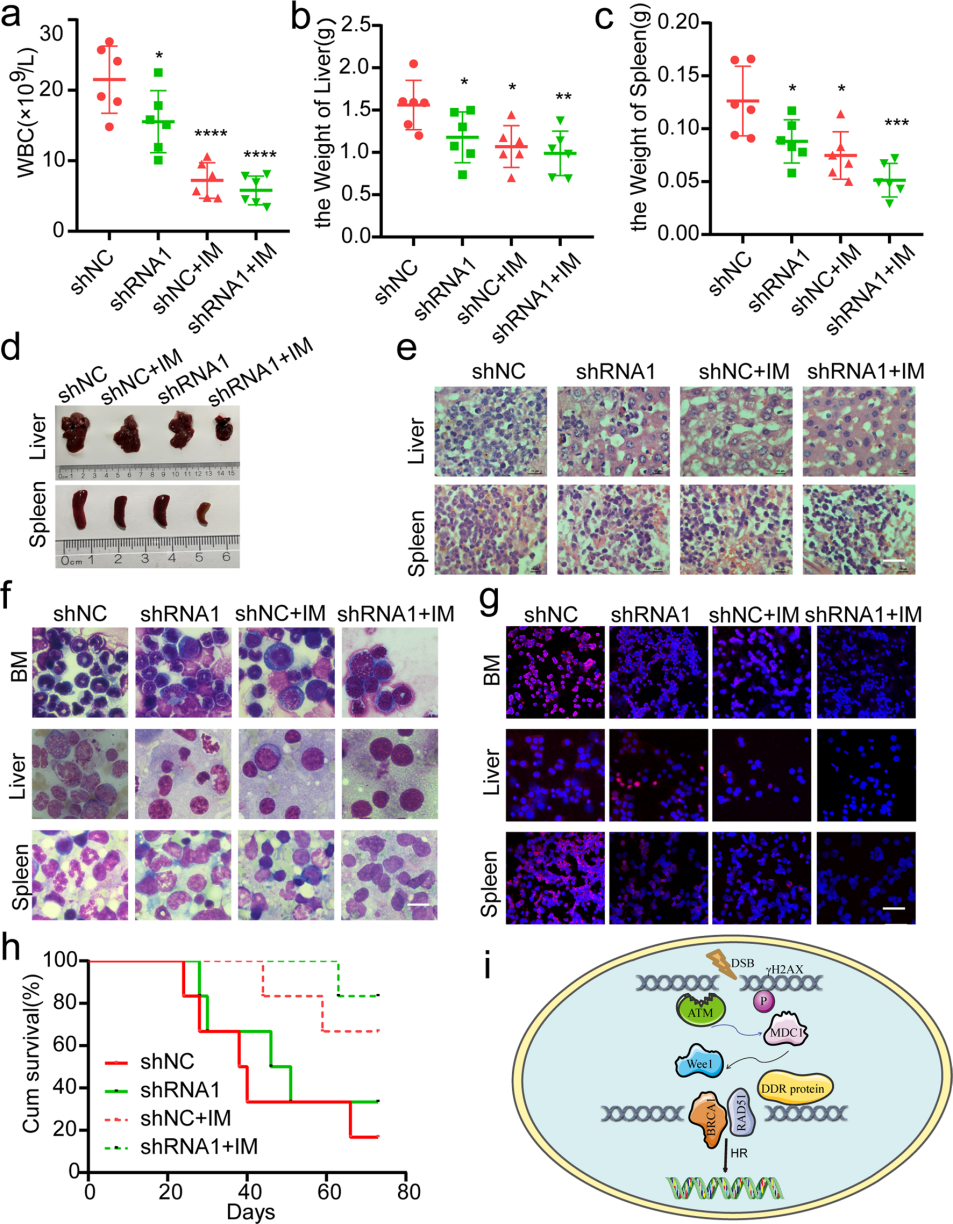
In vivo, the knockdown of Wee1 enhances IM sensitivity. **a** In the mouse model, total WBCs counts were calculated. **b** The livers and **c** spleens weights were calculated. **d** Images of livers and spleens would be displayed. **e**, **f** H&E staining was used to examine liver and spleen infiltration, and the cells of the liver, spleen, and bone marrow were analyzed using Wright’s staining. Scale bar, 10 μm. **g** Immunofluorescence was employed to evaluate the BCR-ABL expression in the liver, spleen, and bone marrow cells. Scale bar, 100 μm. **h** GraphPad 8.0 was utilized to create the survival curves, and Kaplan–Meier technology was used to evaluate them. **i **Pattern diagram of Wee1 involvement in DNA damage response. *p < 0.05, **p < 0.01, ***p < 0.001, ****p < 0.0001

Following that, we examined targeting Wee1 to influence CML cell behavior and chemosensitivity in IM in vivo. NOD/SCID mice were randomly separated into four groups after receiving injections of the K562/G01-shNC and K562/G01-shWee1 cells through tail veins. Seven days after the development of the xenograft models, the mice underwent IM treatment at a therapeutic dosage of 50 mg/kg each time for three weeks (eight times total, i.p.), while the control mice received the same amount of PBS. We sacrificed the mice when they depicted visible symptoms like losing weight, lethargy, erect fur, a hunched back, and an unsteady gait. Mice in the shRNA1 + IM group had lesser white blood cell numbers than mice inside the shNC, shRNA1, and shNC + IM groups (Fig. 8a). Following the removal and weighting of the liver and spleen, the shNC groups of mice showed much greater hepatosplenomegaly than the shRNA1, shNC + IM groups, suggesting that the malignancy manifestations could be alleviated by combining shRNA1 and IM (Fig. 8b–d). Hematoxylin and eosin (HE) staining and Wright’s staining was used to scrutinize the infiltration of leukemic cells in the murine model. Leukemic infiltration was decreased in the shRNA1, shNC + IM xenograft model, particularly in the shRNA1 + IM group, according to the findings (Fig. 8e, f). Results from the immunofluorescent detection of BCR-ABL expression in mouse bone marrow cells, liver, and spleen were comparable to those that had previously been described (Fig. 8g). The shRNA1 + IM group mice also lived a lot longer compared to the other groups (Fig. 8h). Finally, our findings suggest that inhibiting Wee1 may increase CML cell chemosensitivity to IM while also delaying CML malignancy development in vivo, hinting that it might be a therapeutic target for future CML therapy.

## Discussion

Bcr-Abl fusion gene drives the onset and development of CML, whereas the oncoprotein BCR-ABL with increased tyrosine kinase activity serves as a target for TKI treatment [33, 34]. Mutations within the fusion gene are among the various causes of TKI resistance and uncontrolled disease development [32]. Indeed, one of the most prevalent characteristics of cancer, particularly in hematological malignancies, is chromosome instability which primarily results from dysregulation of DNA damage response and repair machinery, and vice versa. According to research, the presence of the fusion protein BCR-ABL increases exaggerated overall DSB capacity, which may result in an unbalanced ratio between DSB ends and the number of repair proteins, as well as a series of dysregulated DNA damage repair pathways to manage the excess genome damage, including the error-prone alternative end-joining [30]. Recent evidence also suggested that DDR signaling pathways serve in the emergence of CML [4, 5], and DDR-related proteins are already being studied as targeted therapies in a variety of diseases [3]. In addition to TKIs, unraveling the proteins implicated and targeting the DDR pathways provide new insights for CML treatment.

This work is the first to shed light on the kinase Wee1’s carcinogenic significance and regulatory mechanism of DDR in CML. Although Wee1 has been previously reported to be an oncogenic factor [19–22], and suppressing Wee1 exerted an anticancer effect in ovarian cancer [35] and osteosarcoma cells [36], whereas in CML, on the other hand, Wee1’s engagement related with DRR has never been extensively illustrated. We reported Wee1’s consistently overexpressed in CML cells when compared to controls cells. It displayed anti-cancer manifestations such as cell proliferation suppression and cell cycle arrest when the aberrant overexpression of Wee1 was knocked down in CML cells. Since Wee1 has been found to connect with DNA damage as a kinase [12, 13], we speculated that Wee1 might be participated in the control of DNA damage in CML to help to exert these anticancer effects. We later discovered that Wee1 knockdown greatly increased unrepaired intracellular DNA damage by studying the relationship between Wee1 and γH2AX. CML cells failed to manage the overwhelming DNA damage and eventually died due to a lack of appropriate and immediate repair pathways.

It’s considered that when DNA double-strand breaks occur, ATM kinase phosphorylates starts an array of downstream effector proteins that perform DNA double-strand break repair, cell cycle control, and cell death, minimizing the risk of cells with unstable genomes replicating [37, 38]. The DNA repair signaling mediator proteins 53BP1, MDC1, BRCA1, and MRE11-RAD50-NBS1 complexes are more likely to be attracted to DNA damage sites when ATM induces the formation of γH2AX foci. When Wee1 was deleted, 53BP1 and MDC1 generated typical DNA damage-induced foci, and the intensity of MDC1 fluorescence increased. These findings indicated that Wee1-depleted cells have broad DDR activation and that Wee1 and MDC1 may work together to respond to DNA damage, although the precise mechanism for controlling DDR remains unknown [29]. We assumed that in CML, Wee1 might collaborate with ATM, γH2AX, and MDC1 to promote subsequent DNA damage repair by responding to DNA damage. As expected, Wee1 was recruited early in DDR, and when we inhibited ATM, the focal accumulation of Wee1 and γH2AX stopped, leading to the expression of Wee1 and MDC1 somewhat decreased. Meanwhile, knocking down MDC1 caused a reduction in Wee1 expression. Therefore, our work showed that Wee1 responds to DNA damage via ATM-γH2AX-MDC1 dependent manner.

The most dangerous type of DNA damage DSBs stimulates two major repair pathways, HR and NHEJ [10, 11]. We demonstrated that Wee1 predominantly influences HR repair when DNA is damaged. Intriguingly, we found that suppressing Wee1 in CML cells reduced the expression of RAD51 and BRCA1. Given that RAD51 and BRCA1 are vital components in homologous recombination repair [31], we reasoned that by interacting with RAD51 and BRCA1, Wee1 may aid to initiate the DNA repair process, preventing DNA damage and enhancing the growth of CML cells. Our findings substantially agree with the idea that Wee1 is rapidly recruited at DSBs in response to DNA damage via ATM-γH2AX-MDC1, driving Wee1 to recruit downstream HR repair proteins BRCA1 and RAD51 to DNA damage sites, as well as allowing cancer cells to escape damage and survive (Fig. 8i). DNA damage repair and response are critical in CML cells, and error-prone DNA repair is among the likely causes of the bcr-abl fusion gene alterations because of its relatively faithless ligation, that also may further results in resistance to BCR-ABL tyrosine kinase inhibitors [32]. Our findings demonstrated that Wee1 was able to influence the overall DSB repair capabilities in CML cells, implying that this kinase may take a part in causing fusion gene bcr-abl alterations and making CML cells resistant to TKIs. However, more evidence of the relationship between DNA damage repair and tyrosine kinase inhibitor resistance is needed.

In summary, the above findings revealed that Wee1 may be a potential strategy for future CML adjuvant therapy. When DSBs occurs in CML cell, the highly expressed Wee1 swiftly reacts to DNA damage via an ATM-γH2AX-MDC1-dependent manner. In addition, it facilitates the hiring of HR-related key proteins RAD51 and BRCA1 to DNA damage sites for repair, therefore it also prevents cancer cells from proliferating and causing cell cycle arrest, which helps them escape the threat and thus survive (Fig. 8i). Inhibition Wee1 can also make CML cells more susceptible to TKIs by accumulating DNA damage.

## Conclusion

We here report that Wee1 reacts to DNA damage in a manner that is ATM-γH2AX-MDC1 dependent and is also implicated in the regulation of the HR repair mechanism by recruiting key repair proteins. Inhibiting abnormally elevated Wee1 benefits CML therapy in both IM-resistant and IM-sensitive cells, manifesting various anti-cancer effects. In the fight against CML, Wee1 is proved to be a promising target.

## Supplementary Information

**Additional file 1: Table 1.** Primer sequences were used in the study.

## Data Availability

Not applicable.

## Abbreviations

CML: Chronic myeloid leukemia
TKIs: Tyrosine kinase inhibitors
IM: Imatinib
DDR: DNA damage response
DSBs: DNA double-strand breaks
ATM: Ataxia-telangiectasia mutated
MDC1: DNA damage checkpoint protein 1
HR: Homologous recombination
NHEJ: Non-homologous end joining
ATR: Ataxia telangiectasia and Rad3 related

## References

[1] Cortes J, Pavlovsky C, Saußele S (2021). Chronic myeloid leukemia. Lancet.

[2] Clark RE (2019). Tyrosine kinase inhibitor therapy discontinuation for patients with chronic myeloid leukaemia in clinical practice. Curr Hematol Malig Rep.

[3] Ghelli Luserna di Rorà A, Cerchione C, Martinelli G, Simonetti G (2020). A WEE1 family business: regulation of mitosis, cancer progression, and therapeutic target. J Hematol Oncol.

[4] Fan Z, Luo H, Zhou J (2020). Checkpoint kinase-1 inhibition and etoposide exhibit a strong synergistic anticancer effect on chronic myeloid leukemia cell line K562 by impairing homologous recombination DNA damage repair. Oncol Rep.

[5] Vakili-Samiani S, Turki Jalil A, Abdelbasset WK (2021). Targeting Wee1 kinase as a therapeutic approach in Hematological Malignancies. DNA Repair (Amst).

[6] Cuadrado M, Martinez-Pastor B, Fernandez-Capetillo O (2006). ATR activation in response to ionizing radiation: still ATM territory. Cell Div.

[7] Rupnik A, Lowndes NF, Grenon M (2010). MRN and the race to the break. Chromosoma.

[8] Khanna KK, Jackson SP (2001). DNA double-strand breaks: signaling, repair and the cancer connection. Nat Genet.

[9] Stewart GS, Wang B, Bignell CR, Taylor AM, Elledge SJ (2003). MDC1 is a mediator of the mammalian DNA damage checkpoint. Nature.

[10] Baumann P, Benson FE, West SC (1996). Human Rad51 protein promotes ATP-dependent homologous pairing and strand transfer reactions in vitro. Cell.

[11] Huertas P (2010). DNA resection in eukaryotes: deciding how to fix the break. Nat Struct Mol Biol.

[12] Do K, Doroshow JH, Kummar S (2013). Wee1 kinase as a target for cancer therapy. Cell Cycle.

[13] Kastan MB, Bartek J (2004). Cell-cycle checkpoints and cancer. Nature.

[14] Boutros R, Lobjois V, Ducommun B (2007). CDC25 phosphatases in cancer cells: key players? Good targets?. Nat Rev Cancer.

[15] O'Farrell PH (2001). Triggering the all-or-nothing switch into mitosis. Trends Cell Biol.

[16] Matsuoka S, Rotman G, Ogawa A, Shiloh Y, Tamai K, Elledge SJ (2000). Ataxia telangiectasia-mutated phosphorylates Chk2 in vivo and in vitro. Proc Natl Acad Sci U S A.

[17] Jazayeri A, Falck J, Lukas C (2006). ATM- and cell cycle-dependent regulation of ATR in response to DNA double-strand breaks. Nat Cell Biol.

[18] Johnson N, Cai D, Kennedy RD (2009). Cdk1 participates in BRCA1-dependent S phase checkpoint control in response to DNA damage. Mol Cell.

[19] Masaki T, Shiratori Y, Rengifo W (2003). Cyclins and cyclin-dependent kinases: comparative study of hepatocellular carcinoma versus cirrhosis. Hepatology.

[20] Iorns E, Lord CJ, Grigoriadis A (2009). Integrated functional, gene expression and genomic analysis for the identification of cancer targets. PLoS ONE.

[21] Tibes R, Bogenberger JM, Chaudhuri L (2012). RNAi screening of the kinome with cytarabine in leukemias. Blood.

[22] Porter CC, Kim J, Fosmire S (2012). Integrated genomic analyses identify WEE1 as a critical mediator of cell fate and a novel therapeutic target in acute myeloid leukemia. Leukemia.

[23] Magnussen GI, Hellesylt E, Nesland JM, Trope CG, Flørenes VA, Holm R (2013). High expression of wee1 is associated with malignancy in vulvar squamous cell carcinoma patients. BMC Cancer.

[24] Music D, Dahlrot RH, Hermansen SK (2016). Expression and prognostic value of the WEE1 kinase in gliomas. J Neurooncol.

[25] Magnussen GI, Holm R, Emilsen E, Rosnes AK, Slipicevic A, Flørenes VA (2012). High expression of Wee1 is associated with poor disease-free survival in malignant melanoma: potential for targeted therapy. PLoS ONE.

[26] Slipicevic A, Holth A, Hellesylt E, Tropé CG, Davidson B, Flørenes VA (2014). Wee1 is a novel independent prognostic marker of poor survival in post-chemotherapy ovarian carcinoma effusions. Gynecol Oncol.

[27] Lal S, Burkhart RA, Beeharry N (2014). HuR posttranscriptionally regulates WEE1: implications for the DNA damage response in pancreatic cancer cells. Cancer Res.

[28] Slipicevic A, Holth A, Hellesylt E, Tropé CG, Davidson B, Flørenes VA (2014). Wee1 is a novel independent prognostic marker of poor survival in post-chemotherapy ovarian carcinoma effusions. Gynecol Oncol.

[29] Domínguez-Kelly R, Martín Y, Koundrioukoff S (2011). Wee1 controls genomic stability during replication by regulating the Mus81-Eme1 endonuclease. J Cell Biol.

[30] Liang Y, Qin Y, Jiang G, Feng W, Yuan Y (2022). Targeting MDC1 promotes apoptosis and sensitizes Imatinib resistance in CML cells by mainly disrupting non-homologous end-joining repair. Med Oncol.

[31] Chappell WH, Gautam D, Ok ST, Johnson BA, Anacker DC, Moody CA (2015). Homologous recombination repair factors Rad51 and BRCA1 are necessary for productive replication of human papillomavirus 31. J Virol.

[32] Fernandes MS, Reddy MM, Gonneville JR (2009). BCR-ABL promotes the frequency of mutagenic single-strand annealing DNA repair. Blood.

[33] Ren R (2005). Mechanisms of BCR-ABL in the pathogenesis of chronic myelogenous leukaemia. Nat Rev Cancer.

[34] de Klein A, van Kessel AG, Grosveld G, Bartram CR, Hagemeijer A, Bootsma D (1982). A cellular oncogene is translocated to the Philadelphia chromosome in chronic myelocytic leukaemia. Nature.

[35] Zhang M, Dominguez D, Chen S (2018). WEE1 inhibition by MK1775 as a single-agent therapy inhibits ovarian cancer viability. Oncol Lett.

[36] Kreahling JM, Gemmer JY, Reed D, Letson D, Bui M, Altiok S (2012). MK1775, a selective Wee1 inhibitor, shows single-agent antitumor activity against sarcoma cells. Mol Cancer Ther.

[37] Bensimon A, Aebersold R, Shiloh Y (2011). Beyond ATM: the protein kinase landscape of the DNA damage response. FEBS Lett.

[38] Blackford AN, Jackson SP (2017). ATM, ATR, and DNA-PK: the trinity at the heart of the DNA damage response. Mol Cell.

