# Supplementary material for: Wee1 promotes cell proliferation and imatinib resistance in chronic myeloid leukemia via regulating DNA damage repair dependent on ATM-γH2AX-MDC1
Source: Cell Commun Signal. 2022 Dec 27;20:199. doi: 10.1186/s12964-022-01021-z (PMC9793686; doi:10.1186/s12964-022-01021-z)
Supplement: Supplementary file 1 — Additional file 1: Table 1. Primer sequences were used in the study. [file 12964_2022_1021_MOESM1_ESM.doc]

Supplementary Table S1 Primer sequences were used in the study.

|  |
| --- |
| Gene Forward/ Reverse Primer Sequence (5'-3') |
| Wee1 F CGCACACGCCCAAGAGTTT  R ACTGGCTTCCATGTCTTCACC |
